# GABAergic synaptic components are largely preserved across human and mouse neuronal models

**DOI:** 10.3389/fncel.2025.1588894

**Published:** 2025-05-02

**Authors:** Beatriz Rebollo, Astghik Abrahamyan, Ulrich-Wilhelm Thomale, Angela M. Kaindl, Melissa A. Herman, Christian Rosenmund

**Affiliations:** ^1^Institute of Neurophysiology, Charité – Universitätsmedizin Berlin, Corporate Member of Freie Universität Berlin and Humboldt-Universität zu Berlin, Berlin, Germany; ^2^NeuroCure Cluster of Excellence, Berlin, Germany; ^3^Pediatric Neurosurgery, Neurosurgical Department, Charité – Universitätsmedizin Berlin, Berlin, Germany; ^4^Department of Pediatric Neurology, Charité – Universitätsmedizin Berlin, Berlin, Germany

**Keywords:** human neurons, GABAergic neurons, synaptic transmission, autaptic culture, neurotransmitter release

## Abstract

Synaptic transmission is essential for brain function. But which characteristics of synapse function are so crucial that they are conserved between species? In general, animal models have shaped our understanding of neuronal function, although in recent years our knowledge of human neurophysiology has vastly increased. Comparative analyses between rodent and human neurons have highlighted the similarities and differences in morpho-electrical features, but the extent to which the properties of neurotransmitter release are conserved is underexplored. In this study, we compared the intrinsic properties that determine synaptic strength in cultured GABAergic neurons from mouse and human. Our findings demonstrate that, while passive neuronal properties are different across species, synaptic properties are similar, suggesting that mechanisms of synaptic transmission are conserved between mouse and human neurons. This work provides valuable insight into the extent to which animal models reflect human synaptic components at the single cell level.

## Introduction

Synaptic transmission is essential for information-processing in the brain. It supports neuronal communication and plays a critical role in neurodevelopment and higher-order cognitive functions. Pathophysiological alterations of synaptic transmission are thought to be the primary cause of many neurological disorders (Verhage and Sorensen, 2020). However, the molecular mechanisms underlying synaptic impairment are poorly understood due to the complexity and inaccessibility of the human brain. Part of this complexity relies in the vast diversity of cell types that form neuronal networks. The two main cell types are excitatory and inhibitory neurons, which release glutamate or gamma-aminobutyric acid (GABA) neurotransmitter, respectively, at their synapses. Recent advances in molecular technologies have enhanced our understanding of the diversity of these two neuronal types in the human brain, as well as facilitated comparative analysis across species (Szegedi et al., 2020; Bakken et al., 2021; Kalmbach et al., 2021; Chartrand et al., 2023), resulting in a cross-species consensus of neuronal types at the transcriptomic and physiological level. Additionally, recent studies using multi-neuron patch-clamp recording in acute human brain slices have shown a more heterogenous connectivity in the human cortex compared to the mouse cortex, suggesting that complex synaptic events may contribute to superior cognitive abilities in humans (Testa-Silva et al., 2014; Szegedi et al., 2016; Peng et al., 2019; Campagnola et al., 2022; Planert et al., 2023; Peng et al., 2024). These studies showed differences in synaptic connectivity between human and mouse at the network level, but the cell-specific synaptic properties underlying such differences were not addressed. Newly developed methods in human organotypic slice culture have provided further insight into the connectivity of principal glutamatergic neurons and GABAergic interneurons in the human cortex, even revealing target-specific short-term plasticity dynamics in glutamatergic to interneuron subtype synaptic connections (Kim et al., 2023). Yet, a detailed analysis of neurotransmitter release characteristics from human synapses, particularly those in GABAergic neurons, are still warranted.

Non-human animal models have been crucial in describing the process of neurotransmitter release, such as defining the “quantal hypothesis” in which neurotransmitters are released as quanta or molecular packages (Fatt and Katz, 1952). In this regard, quantification of quantal parameters is fundamental to determining synaptic transmission efficacy. This has been extensively studied in *in vitro* animal models with optical and electrophysiological methods (Rosenmund and Stevens, 1996; Pulido et al., 2015; Bekkers, 2020; Durst et al., 2022). However, studies of cell-specific synaptic properties in human brain tissue slices, have focused on circuit connectivity without providing a detailed quantal analysis of neurotransmitter release (Peng et al., 2019; Campagnola et al., 2022; Kim et al., 2023; Planert et al., 2023). A reliable approach that can be generalized to different neuronal models is required in order to quantitatively characterize synaptic efficacy in human neurons and compare it to that described in animal models.

Neurons derived from human induced pluripotent stem cells have opened new avenues for studying human neuronal function (Marchetto et al., 2010; Mori et al., 2024). Adapting the specialized experimental culture system of single neurons growing on astrocytic feeder layer microislands, known as autapses, to the human induced neurons (hIN) model has enhanced our understanding of synaptic transmission characteristics in human neurons (Fenske et al., 2019; Meijer et al., 2019; Rhee et al., 2019). These autaptic cultures, in which neurons make synapses only onto themselves, provide a reliable model to study synapse formation and function (Meijer et al., 2019; Rhee et al., 2019). However, how synaptic transmission studied in autaptic cultured hINs compares to autaptic neurons cultured directly from human is not yet known. The combined electrophysiological, morphological and transcriptomic analysis of hINs in different cellular states at the single-cell level revealed a continuum of functional states related to different maturation points and provided evidence for functional heterogeneity of hINs (Bardy et al., 2016). Nonetheless, it is unclear exactly the maturation state that hINs represent with regard to human development. Therefore, a direct comparison of hINs to human neurons dissociated and cultured from brain tissue is necessary to get insight into the extent to which physiological properties are conserved when human neurons are induced *in vitro*.

In this study, we provide a detailed characterization of the intrinsic properties that determine synaptic strength in human neonatal GABAergic neurons grown in primary culture, and directly compare it to cultured hINs and mouse neurons. We adapted the autaptic culture system to primary cortical human neurons (cHN), induced human neurons (iHN), primary cortical mouse neurons (cMN) and, primary striatal mouse neurons (sMN) to analyze synaptic transmission efficacy across models and species. Using electrophysiological recordings together with immunolabeling, we quantified the quantal parameters of GABA release from inhibitory synapses. We found that the main synaptic components, such as postsynaptic responses, number of vesicles available for release and vesicle release probability, were similar between cHN and cMN. iHN showed reduced synaptic responses compared to the primary neuronal cultures, indicative of a more immature state. This work demonstrates the side-by-side comparison of synaptic mechanisms across species and cell types using autaptic cultures. Overall, our comparative approach provides a basis for future studies that utilize mouse models to understand the extent to which such models reflect human neuronal function.

## Materials and methods

### Human tissue acquisition

Human neurons were obtained from parasagittal transventricular hemispherotomy in one patient (1-week old) suffering from drug-resistant lesional epilepsia (Makridis et al., 2021). The study procedures adhered to ethical requirements and were approved by the local ethical committee with Approval Nr. EA2/084/18. Prior written informed consent for the scientific use of resected tissue was given by the parents.

### Generation of induced GABAergic neurons

IPSCs were obtained from an iPSC line provided by the Berlin Institute of Health Core Facility Stem Cells (Germany): The BIHi005-A iPSC line.^1^ IPSCs were maintained in StemFlex medium (ThermoFisher Scientific). Inhibitory human induced neurons were generated as described previously (Yang et al., 2017). Briefly, 1 day before induction, iPSCs were dissociated with Accutase cell dissociation reagent (ThermoFisher Scientific), plated on Matrigel (Corning) -coated plates containing StemFlex medium supplemented with 2 μM thiazovivin (Tocris), and infected with lentiviral vectors containing doxycycline-inducible Ascl1 and Dlx2, fluorescent reporter and puromycin resistance (FUW-TetO-DLX2.P2A.AsclI.IRES.Puro, FUW-eGFP and FUW-rtTA, respectively). Lentiviral particles were produced by the Charité Viral Core Facility as previously described (Fenske et al., 2019).

On day 0, the medium was changed to DMEM/F12 (ThermoFisher Scientific) containing N-2 supplement (ThermoFisher Scientific), and doxycycline (2 μg/ml, Sigma Aldrich) to induce TetO-dependent gene expression. On day 1 and 2, DMEM/F12 medium was replaced and 0.5 mg/l puromycin to select for induced iNs. On day 3, medium was changed to DMEM/F12 medium containing cytosine β-D-arabinofuranoside (Ara-C, 4 μM) to avoid proliferation of non-neuronal cells. On day 6, induced neurons were reseeded onto new Matrigel/Poly-ornitin/Laminin coated plates together with mouse glia cells and maintained in growth medium containing: Neurobasal-A Medium (NBA, SigmaAldrich) with B-27 supplement, GlutaMAX, doxycycline (2 μg/ml), 5% fetal bovine serum (PanBiotech), penicillin (100 U/ml) and streptomycin (100 μg/ml) (ThermoFisher Scientific). On day 9, half of the medium was replaced and Ara-C added to avoid proliferation of glia cells. On day 14, half of the growth medium was changed to NBA with B-27 supplement, GlutaMAX, BDNF 10 ng/ml, Ara-C (2 mg/ml), 5% fetal bovine serum (PanBiotech), penicillin (100 U/ml) and streptomycin (100 μg/ml). From day 17 on, half of the medium in each well was replaced every 5 days with growth medium plus Ara-C and BDNF. After 70–90 days, cells were washed twice with PBS (ThermoFisher Scientific) and dissociated with Accutase for re-spliting and plating onto astrocyte micro-islands.

### Mouse

Animals were used in accordance with the regulations established by the Animal Welfare Committee of Charité Medical University and the Berlin State Government Agency for Health and Social Services (Lisence T0220/09). Newborn mice (P0-2) from VGAT-venus mouse line, in which GABAergic neurons are fluorescently labeled, were used (Wang et al., 2009).

### Cell culture

Human and mouse neurons were seeded and cultured on micro-island astrocyte feeder layers as described previously (Paraskevopoulou et al., 2021). Neurons were plated at a density of 3.5–6K, per 35-mm-diameter dish and grown in a defined medium (NBA with B27, GlutaMax, and penicilin/streptromycin; Invitrogen) for 2 weeks. In the case of cortical human cultures 2.5% fetal calf serum (FCS) was added. Experiments were performed at 13–15 days *in vitro* (DIV).

### Electrophysiology

Whole-cell patch-clamp recordings were performed on autaptic neurons using a Multiclamp 700B amplifier (Molecular Devices) controlled by Clampex 10.7 software (Molecular Devices). Data were digitally sampled at 10 kHz with an Axon Digidata (1440A digitizer, Molecular Devices) and low-pass Bessel filtered at 3 kHz. Neurons were constantly perfused with an extracellular solution containing the following (in mM): 140 NaCl, 2.4 KCl, 10 HEPES, 10 glucose, 2 CaCl_2_ and 4 MgCl_2_ (∼300 mOsm, pH 7.4). Recordings were performed with borosilicate glass pipettes (2–4 MΩ) filled with the following internal solution (in mM): 136 KCl, 17.8 HEPES, 1 EGTA, 4.6 MgCl_2_, 4 Na_2_ATP, 0.3 Na_2_GTP, 12 creatine phosphate, and 50 U/ml phosphocreatine kinase (∼300 mOsm, pH 7.4). All experiments were performed at room temperature.

Membrane capacitance was measured from the membrane test function in pClamp (Molecular Devices). For current-clamp recordings, a current step of –50 pA for 300–600 ms was injected, from which resting membrane potential was measured and input resistance was calculated by Ohm’s law. The membrane time constant (tau) was obtained by fitting an exponential to the voltage response to the −50 pA current pulse.

For voltage-clamp recordings, neurons were held at −70 mV potential. IPSCs were evoked by unclamped action potential elicited by a 2 ms depolarizing pulse to 0 mV. The decays of IPSCs or miniature IPSCs (mIPSCs) were fitted by one- or two-phase exponentials with the best fit being determined (GraphPad, Prism). A weighted decay was computed for two-phase exponential fittings. RRP was determined by applying a hypertonic sucrose solution (500 mM) for 5 s and quantified by integrating the transient component (Rosenmund and Stevens, 1996). The number of vesicles in the RRP was calculated by dividing the sucrose charge by the charge of the average mIPSC. To detect individual mIPSC events, data were filtered at 1 kHz, and detected with a template (0.5 ms rise, 18 ms decay) above the threshold of 3 times the SD. For PPR, IPSCs were recorded as interleaved single and paired pulses (20 or 10 Hz, as indicated). The IPSC amplitude for pulse 1 was measured from a baselined average of several single traces. The IPSC for pulse 2 was measured from the baselined average paired-pulse trace minus the baselined average interleaved single pulse trace. PPR was calculated by dividing the IPSC amplitude of pulse 2 by the IPSC amplitude of pulse 1. To quantify vesicle release probability a single IPSC was evoked 3 s before the sucrose application (IPSC_suc_). Pvr was calculated as the ratio of the IPSC_suc_ charge over the RRP charge.

### Immunocytochemistry

Neurons were fixed in 4% w/v paraformaldehyde and prepared for immunocytochemistry as previously described (Paraskevopoulou et al., 2019). Specifically, primary antibodies dilutions were as follows: chicken anti-microtubule-associated protein 2 (MAP2) (1:2000, Millipore), rabbit anti-vesicular GABA transporter (VGAT) (1:1000, Synaptic Systems).

Images were acquired using either an Olympus IX81 inverted epifluorescence microscope with a CCD camera (Princeton MicroMay; Roper Scientific, Trenton, NJ) and MetaMorph software (Molecular Devices), or on a Leica SP8 laser-scanning confocal microscope. Quantification of neuronal morphology (dendrite length and soma area) was performed by tracing MAP2-positive processes with NeuronJ plugin (Meijering et al., 2004) and area across the MAP2-positive cell body was measured to estimate the soma area. Dendrite thickness was quantified at a distance of 30 μm from the soma, using dendrite length of 25–30 μm. Synapse number was identified by immunoreactivity to VGAT antibody, and quantify by VGAT-positive fluorescence puncta. Background was subtracted with a rolling ball of a 30 pixels radius and threshold adjustment. After, images were converted to binary using ImageJ plugin and VGAT puncta were determined using the analyze particle function. Only puncta with 0.01–6.01 pixels^2^ was included in the analysis.

### Analysis and statistics

Current-clamp and voltage-clamp electrophysiological analyses were performed with Clampfit and AxoGraph software, respectively. Fiji software was used for imaging analysis. Raw values were exported to GraphPad Prism for further statistical analysis. To test for normal distribution of the data we used Kolmogorov-Smirnov test, and statistical significance was determined by Kruskal-Wallis test coupled with Dunn’s *post hoc* test.

## Results

### Passive membrane properties of neurons

**FIGURE 1:**
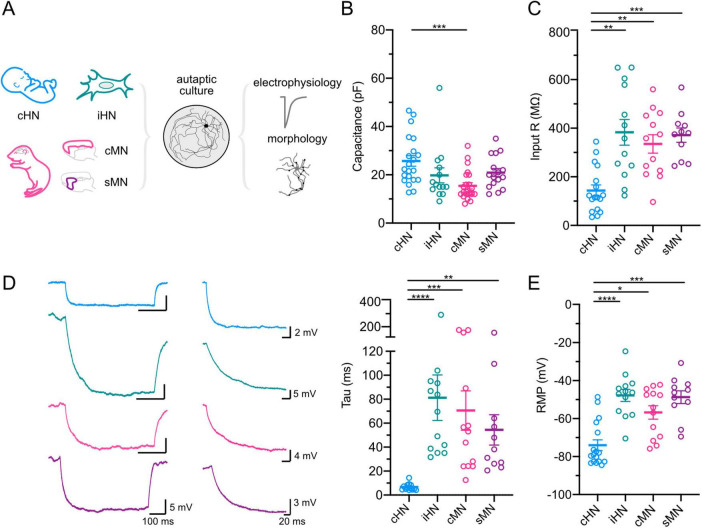
Passive membrane properties of GABAergic neurons. **(A)** Schematic of neuronal models and processing pipeline. **(B–E)** Scatter-plots showing mean capacitances **(B)**; input resistances **(C)**; membrane tau constants, with representative voltage traces in response to hyperpolarizing current pulses (left), and amplification of the first 200 ms period of the voltage traces showing the decay time constant (middle) **(D)**; and resting membrane potentials **(E)**. Data shown as mean ± SEM. Kruskal-Wallis test: *p ≤ 0.05, **p ≤ 0.01, ***p ≤ 0.001, and ****p ≤ 0.0001.

How do passive membrane properties of human and mouse neurons (Mohan et al., 2015; Chartrand et al., 2023; Lee et al., 2023) manifest in autaptic culture? We examined intrinsic membrane properties of GABAergic neurons from our four models using whole-cell voltage- and current-clamp recordings. Cortical HN showed the largest capacitance, significantly larger than the capacitance of cMN (*p* = 0.0003) (Figure 1B; Supplementary Table 1). This suggests that cHN has a larger surface area, potentially accommodating a greater number of ion channels and leading to higher membrane conductance. To quantify input resistance and membrane time constant (tau), we applied hyperpolarizing pulses. Consistent with large capacitance measurements, cHN elicited the lowest input resistance compared to iHN, cMN, and sMN (*p* = 0.001; *p* = 0.0072; *p* = 0.0008, respectively) (Figure 1C). In accordance with a low input resistance, the membrane time constant (tau) of cHN was 12-8 times faster than the taus of iHN, cMN, and sMN (*p* < 0.0001; *p* = 0.0001; *p* = 0.0017, respectively) (Figure 1D). Finally, cHN showed the most hyperpolarized resting potentials, significantly more negative than iHN, cMN, and sMN (*p* < 0.0001; *p* = 0.0183; *p* = 0.0003) (Figure 1E; Supplementary Table 1). This could suggest differences in the composition of voltage-gated channels. Overall, the passive membrane properties of GABAergic cHN were significantly different from GABAergic iHN, cMN and sMN suggesting model- and species-specific divergence.

### Comparison of the magnitude of synaptic transmission across four models

**FIGURE 2:**
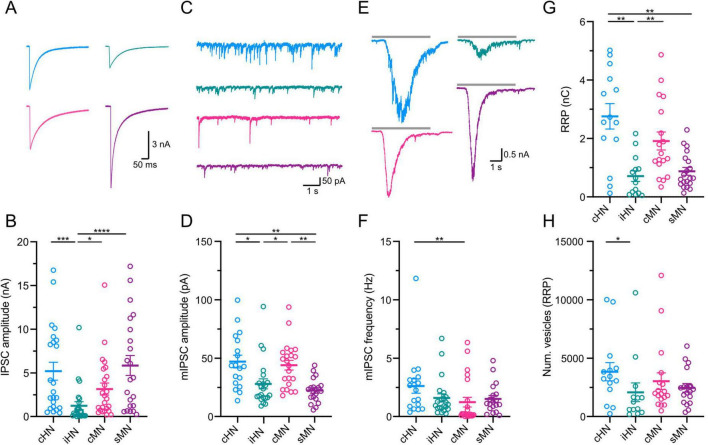
Characterization of GABAergic synaptic transmission. **(A)** Representative traces of evoked IPSCs. Stimulation artifacts were removed from the traces for illustrative purpose. **(B)** Scatter-plot showing mean IPSC amplitudes. **(C)** Representative trace of mIPSC activity. **(D)** Scatter-plot showing the amplitudes of mIPSC. **(E)** Representative current traces of the response to a 5 s hypertonic sucrose solution pulse. **(F)** Scatter-plot showing frequency of mIPSC. **(G)** Scatter-plot showing mean RRP amplitudes. **(H)** Scatter-plot showing number of vesicles. Data shown as mean ± SEM. Kruskal-Wallis test: *p ≤ 0.05, **p ≤ 0.01, ***p ≤ 0.001, and ****p ≤ 0.0001.

Knowing that human and mouse neurons differ in their intrinsic properties, we wonder whether differences also exist in synaptic transmission efficacy. We first investigated the magnitude of action potential-evoked responses by recording inhibitory postsynaptic currents (IPSCs) upon the induction of an unclamped action potential in individual cultured autaptic neurons from each model. iHN elicited the smallest IPSC with an average amplitude of 1.25 ± 0.45 nA (*n* = 24) (Supplementary Table 1), which significantly differed from the other models (cHN: *p* = 0.0003; cMN: *p* = 0.0185; sMN: *p* < 0.0001) (Figures 2A, B), potentially indicating that the iHN form the smallest number of synapses during this period. To gain further insight into the characteristics of the postsynaptic responses in the four models, we compared the rise and decay time constant of the IPSCs (Supplementary Figures 1A, B). No significant differences in rise time were observed across the models. The resulting weighted IPSC decay time constant of cHN was almost two-fold faster than the decay time of iHN, cMN and sMN, pointing to differences in the composition of postsynaptic receptors.

#### Quantification of parameters underlying synaptic transmission

The magnitude of the response elicited by an action potential is the product of three components of the synapse: the quantal size (q), the number of vesicles available to participate in neurotransmitter release or readily-releasable pool (RRP) (n), and the probability by which neurotransmitter is released from a synaptic vesicle (p). We compared each of these underlying components of synaptic transmission across the four experimental neuronal models.

To estimate quantal size, we recorded miniature IPSCs (mIPSCs) (Figure 2C and Supplementary Figure 1C). The average mIPSC amplitude was not different between cHN and cMN. However, iHN and sMN showed mIPSC amplitudes that were half of that observed in cHN and cMN (cHN vs iHN: *p* = 0.0131; iHN vs cMN: *p* = 0.0169; cHN vs sMN: *p* = 0.0016; cMN vs sMN: *p* = 0.0020) (Figure 2D; Supplementary Table 1). Also, cHN and cMN showed similar mIPSCs charge values that were ∼40% larger than that in iHN and sMN (Supplementary Figures 1C, D). cHN showed significant slower mIPSC rise time compared to the other models (cHN vs iHN: *p* = 0.002; cHN vs cMN: *p* = 0.0007) (Supplementary Figure 1E). No differences in mIPSC decay time were observed across the groups (Supplementary Figures 1D, F). The frequency of mIPSCs was twice as high in cHN compared to cMN (*p* = 0.0026), while iHN and sMN showed similar mIPSC frequencies to cMN (Figure 2F; Supplementary Table 1).

We next quantified the readily-releasable pool (RRP) of synaptic vesicles (see section “Materials and methods”) in cHN, iHN, cMN, and sMN autaptic cultures using hypertonic sucrose (Rosenmund and Stevens, 1996). The average RRP charge was similar between cHN and cMN GABAergic neuron models, while iHN elicited the smallest RRP charge (Figures 2E, G; Supplementary Table 1). This is consistent with the smaller amplitude IPSC observed in iHN, indicating a smaller number of synapses formed by neurons in this model. Next, by dividing the RRP charge by the average charge of the mIPSCs, we calculated the number of vesicles in the RRP (Figure 2H). This measurement was similar among cHN, cMN and sMN, whereas iHN had the lowest number of vesicles (cHN vs iHN: *p* = 0.0408) (Supplementary Table 1). The differences in vesicle number were also comparable with the differences observed in IPSC amplitude, reflecting the relationship between evoked IPSC magnitude and the number of fusion competent vesicles.

**FIGURE 3:**
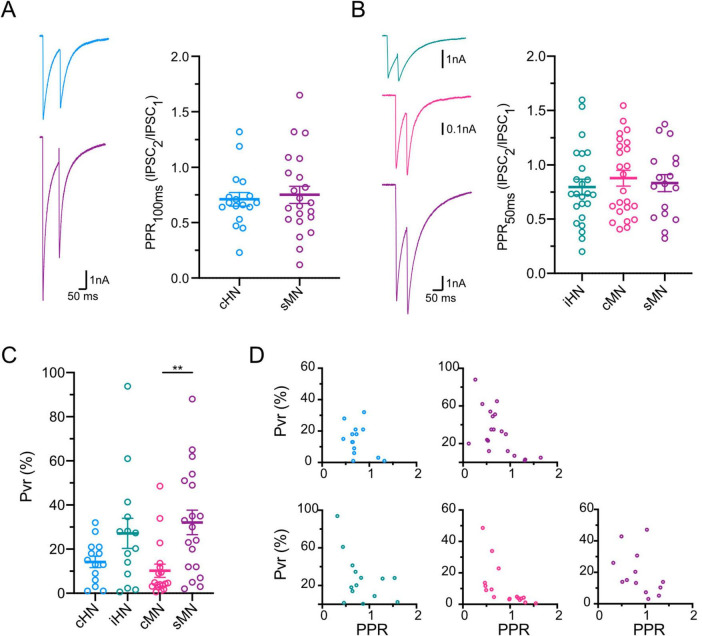
Characterization of GABAergic synaptic plasticity. **(A)** Representative traces of inhibitory responses to paired pulse stimulation with 100 ms interstimulus interval for cHN and sMN, left; scatter-plot showing paired pulse ratios, right. **(B)** Same as A for paired pulse stimulation with 50 ms interstimulus interval for iHN, cMN and sMN. Stimulation artifacts were removed from the traces for illustrative purpose. **(C)** Scatter-plot showing vesicular release probabilities. **(D)** Correlation plots of PPR vs Pvr for each neuronal model; 100 ms ISI, top; 50 ms ISI, bottom. Data shown as mean ± SEM. Kruskal-Wallis test: **p ≤ 0.01.

Finally, we investigated the probability by which neurotransmitter is released in each model. The probability of a single vesicle to be released (Pvr) is quantified in autaptic cultures by dividing the charge of the IPSC by the charge of the RRP (see section “Materials and methods”). Synapses from cHN and cMN contained vesicles with a similar Pvr, while Pvr estimates for iHN and sMN were twice as high (Figure 3C; Supplementary Table 1). In addition, synaptic release probability is reflected in short-term presynaptic plasticity dynamics (Regehr, 2012). Therefore, we calculated paired-pulse ratio (PPR) (see section “Materials and methods”). We did not perform a direct statistical comparison of average PPR among groups with different inter-stimulus interval values; however, our data showed qualitatively similar levels of depression among the four models regardless of the inter-stimulus interval (Figures 3A, B; Supplementary Table 1), indicative of a high release probability at these synapses. As expected, we observed the negative correlation between Pvr and PPR values for each model, where neurons with higher Pvr tended to have more depression during paired-pulse stimulation (Figure 3D). Overall, the similarities between cHN and cMN observed in the quantal parameters that determine synaptic strength (i.e., quantal size or mIPSC amplitude, RRP and Pvr) indicate that synaptic transmission mechanisms of cortical inhibitory neurons are preserved across species.

### Morphological comparison validates the similarities of synaptic transmission across models

Thus far, our data suggest a greater release efficiency in cHN and cMN mediated by a larger number of readily-releasable vesicles. To investigate whether this is related to more synapses or a higher number of readily-releasable vesicles per synapse we performed morphological analysis. Specifically, soma area, dendritic length and thickness were measured with MAP2 labeling, and GABAergic synapses were marked with immunolabeling of the vesicular GABA transporter, VGAT.

**FIGURE 4:**
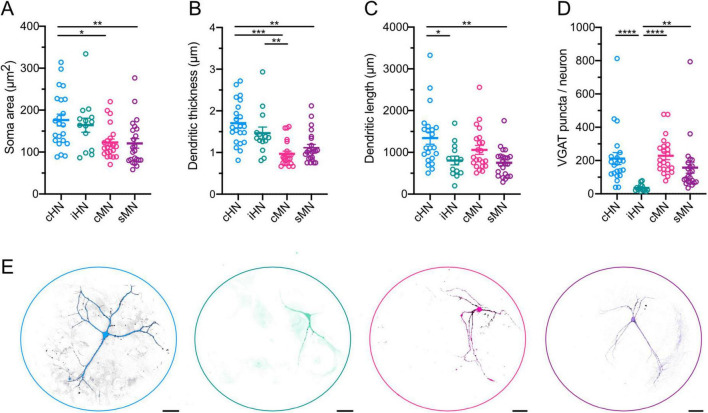
Morphological characterization of GABAergic neurons. **(A–D)** Scatter-plot showing mean soma areas **(A)**, dendritic thickness **(B)**, dendritic lengths **(C)**, and VGAT synapse puncta **(D)**. **(E)** Representative images of neuronal morphology showing immunostaining for MAP2 (color coded) and VGAT (black). Scale bars, 40 μm. Data shown as mean ± SEM. Kruskal-Wallis test: *p ≤ 0.05, **p ≤ 0.01, ***p ≤ 0.001, and ****p ≤ 0.0001.

The average soma area of cHN was ∼45% larger than that of cMN and sMN, but similar to iHN soma area (Figure 4A; Supplementary Table 1). These findings are consistent with the differences observed in the capacitance and indicative of species-specific divergence (Figure 1). Also, dendritic thickness was significantly different between human and mouse models (cHN vs cMN: *p* < 0.0001; cHN vs sMN: *p* = 0.0019; iHN vs cMN: *p* = 0.007) (Figure 4B). cHN showed the largest dendritic length, which was comparable to cMN and significantly different from iHN and sMN (*p* = 0.043 and *p* = 0.0029, respectively) (Figure 4C; Supplementary Table 1). The more expanded dendrites of cHN and cMN could facilitate the formation of more synapses in these models. To explore this, we quantified the number of VGAT puncta.

The total number of VGAT-positive puncta detected in autaptic cultures represents the total number of synapses formed by each individual GABAergic neuron. We found that the number of VGAT-positive puncta was similar between cHN and cMN (Figure 4D; Supplementary Table 1), which further corroborates that synapse formation mechanisms of cortical inhibitory neurons are preserved across species. Moreover, the iHN model exhibited the lowest number of puncta (iHN vs cHN: *p* < 0.0001; iHN vs cMN: *p* < 0.0001; iHN vs sMN: *p* = 0.0021), which aligns with the smallest amplitude IPSC, the smaller RRP, and the fewest readily-releasable vesicles reported above (Figures 2B, G, H). This supports the notion that the iHN model forms a smaller number of synapses in comparison with the other models.

### Discussion

Synaptic transmission is driven by complex mechanisms, the comprehension of which requires studies with animal models. However, comparative analysis is necessary to generalize the findings from animal models to human. Here, using whole-cell patch-clamp recordings and immunocytochemistry we have characterized intrinsic membrane properties, and fundamental synaptic features that define synaptic strength, on GABAergic autaptic human neurons, and directly compare it to GABAergic iHN, cMN and sMN.

Our data show one of the major differences between human and mouse neurons is intrinsic membrane properties (Figure 1). These results are in line with previous studies describing the differences in the intrinsic properties and subcellular architecture of cortical GABAergic neurons across species (Mohan et al., 2015; Chartrand et al., 2023; Lee et al., 2023). However, it should be noted that the input resistance of cHN is significantly lower than that of iHN and mouse neurons (Figure 1C). The fast membrane time constant observed in cHN neurons could indicate that temporal summation of postsynaptic currents *in vivo* may only occur within a narrow time window in human neurons. However, our findings in cultured neurons are in contrast with a recent publication where higher input resistance was measured in adult human GABAergic neurons compared to mouse in acute slice (Lee et al., 2023). Therefore, the intrinsic membrane properties we observed in the cultured neonatal human GABAergic neurons could be a result of immaturity or lack of network input. Nevertheless, the lower input resistance together with the faster membrane time constant and lower RMP observed in cHN suggest that these cells have a different ion channel composition. Whether this is due to the age or the human GABAergic neurons or because they have been grown in culture could not be determined. Single-cell sequencing has related electrophysiological differences between human and mouse neurons to differences in channel composition (Zeng et al., 2012; Chartrand et al., 2023). For instance, it has been shown that human neurons express more hyperpolarization-activated h-channels than mouse (Kalmbach et al., 2018). Activation of these channels at hyperpolarized potentials could contribute to the low input resistance and faster membrane time constant. Inward rectifying potassium channels (Kir), which bring the resting membrane potential closer to the potassium equilibrium (Isomoto et al., 1997), could contribute to a more hyperpolarized resting potential and lower input resistance in the cHN model, though whether these channels are more abundantly expressed in human neurons is unknown.

The results from iHN showed IPSC amplitudes similar to those reported by previous studies at a similar time point (Meijer et al., 2019; Rhee et al., 2019), with our direct comparison to IPSC amplitudes from cultured cHN and mouse neurons confirming significant differences that were not previously reported (Figures 2A, B). These differences in the magnitude of IPSC may be related to different maturation stages. Because iHN develop from a neural stem cell-like phenotype, it is likely that our iHN are at an earlier developmental stage at 104 days post-induction, and the lower IPSC amplitude reported here is due to slower iHN maturation. The three alternative models, cHN, cMN, sMN were all derived from postnatal subjects. Additionally, the induction protocol used to derive GABAergic neurons from pluripotent stem cells is a simplified version of the complex developmental processes occurring in neuronal stem cells *in vivo* (Yang et al., 2017). Therefore, the induced GABAergic neurons may lack other more sophisticated chronological or network-activity-dependent maturation programs. Further studies at different time points will help to identify the corresponding developmental stages across models.

Based on the quantal hypothesis, the strength of neurotransmitter release is the product of quantal parameters – quantal size, release probability and the number of readily-releasable vesicles. Here, taking advantage of the autaptic culture system, we provide a comprehensive comparison of quantal parameters across four different models. In particular, the underlying components of synaptic transmission in cHN and cMN display largely conserved characteristics, with strong similarities in spontaneous release events, RRP and Pvr (Figures 2C–H, 3C). Overall, our data show a high degree of variability, which may reflect the diversity of GABAergic cell types present in cortical neuronal networks (Gouwens et al., 2020). The Pvr variability may be a result of the heterogeneity of release site related to specific cell types in cHN, cMN and sMN; while it may indicate different functional maturation stage in iHN (Bardy et al., 2016). More evidence of preserved synaptic mechanisms across species is based on the IPSC decay kinetics (Supplementary Figure 1A), as a double exponential fit the data better in all four models, indicating the conservation of both Ca^2+^-dependent synchronous and asynchronous neurotransmitter release (Goda and Stevens, 1994). This provides a reliable foundation to make inferences about presynaptic functions from studies using animal models where access and manipulation of human tissue is a limiting factor.

Neurons have a unique structure where neuronal arborization plays an active role in their function. A comparison of soma area, dendritic length and VGAT-positive puncta across the four autaptic models (Figure 4) allows us to further evaluate the morphological aspects of synaptic transmission, and to validate the ideas postulated on the basis of the physiological properties. On one hand, the observed differences in soma area between cHN and cMN indicate species-specific differences that are related to differences in intrinsic membrane properties (Figure 1). On the other hand, the similarities in dendritic length and VGAT puncta between cHN and cMN indicate that the mechanisms underlying synapse formation are preserved across species, as suggested by fundamental synaptic components like mIPSC amplitude and RRP (Figure 2). While more detailed comparisons of dendritic branching and axonal expansion have been reported (Chartrand et al., 2023; Lee et al., 2023), the constraint of our autaptic model, where a single neuron grows on a defined 200 μm diameter astrocytic island, reveals the similarity between intrinsic growth programs cortical mouse and human neurons in a highly controlled environment.

In cortex, there are many types of transcriptionally and functionally distinct interneurons subtypes (Gouwens et al., 2020), and striatal neurons mostly include medium spiny neurons, which are GABAergic primary output neurons (Gokce et al., 2016). Nonetheless, for our analysis, we have generalized GABAergic neurons, without breaking them into further subtypes. This is for several reasons. First, the limited access to newborn human samples only allowed us to perform one set of experiments where we identified GABAergic neurons. Further, synaptogenesis (Chang et al., 2014; Paraskevopoulou et al., 2019) and potentially refinement of cell type identity (Fishell and Kepecs, 2020) are influenced by electrical activity with inhibitory synapse formation particularly affected by glutamatergic activity, a component that is missing in the autaptic system we used. The functional simplicity of autaptic cultures provides a reliable model to study synaptic strength that applies to every GABAergic neuron regardless of its origin and identity. In particular, primary human neuronal cultures can serve to investigate cell intrinsic mechanisms that determine synaptic strength in human neurons and, ultimately, define circuit dynamics and higher order cognitive processes. Overall, our comparative approach enhances our understanding about the synaptic properties that are conserved between human and mouse models, providing insights into fundamental principles of neurotransmitter release. This will help future studies that utilize rodent disease models to understand the extent to which such models reflect synaptopathies in humans.

## Data Availability

The original contributions presented in this study are included in this article/Supplementary material, further inquiries can be directed to the corresponding authors.

## Footnotes

1
https://hpscreg.eu/cell-line/BIHi005-A

## Supplementary material

Supplementary Table 1 Average values for all measured parameters.

Supplementary Figure 1 Kinetic characterization of GABAergic synaptic transmission. **(A)** Scatter-plot of IPSC decay times. **(B)** Scatter-plot showing mIPSC rise times. **(C)** Scatter-plot showing mean mIPSC charges. **(D)** Representative averaged traces of mIPSC. **(E)** Scatter-plot showing mIPSC rise times. **(F)** Scatter-plot showing mIPSC decay times. Pie-plot insets represent number of neurons fitting to single (solid) or double (empty) exponential decay function. Data shown as mean ± SEM. Kruskal-Wallis test: **p* ≤ 0.05, ***p* ≤ 0.01, ****p* ≤ 0.001, and *****p* ≤ 0.0001.
