# Supplementary material for: GABAergic synaptic components are largely preserved across human and mouse neuronal models
Source: Front Cell Neurosci. 2025 May 2;19:1588894. doi: 10.3389/fncel.2025.1588894 (PMC12082711; doi:10.3389/fncel.2025.1588894)
Supplement: Supplementary Table 1 — Average values for all measured parameters. [file Data_Sheet_1.pdf]

Table S1.

	cHN	iHN	cMN	sMN
Capacitance (pF)	25.74 ± 2.19<br>n = 21	19.79 ± 3.12<br>n = 14	15.44 ± 1.26<br>n = 23	20.93 ± 1.69<br>n = 16
Input R (MΩ)	143.5 ± 22.83<br>n = 17	383.1 ± 52.70<br>n = 13	335.3 ± 37.96<br>n = 13	371.4 ± 29.26<br>n = 11
Tau (ms)	6.64 ± 0.80<br>n = 13	81.22 ± 19.04<br>n = 13	70.55 ± 16.43<br>n = 13	54.42 ± 12.63<br>n = 11
RMP (mV)	-74.03 ± 2.84<br>n = 17	-47.77 ± 3.15<br>n = 13	-56.77 ± 3.53<br>n = 13	-48.71 ± 3.36<br>n = 11
IPSC (nA)	5.20 ± 1.02<br>n = 23	1.25 ± 0.45<br>n = 24	3.17 ± 0.68<br>n = 25	5.85 ± 1.13<br>n = 22
IPSC decay time (ms)	47.03 ± 3.05<br>n = 21	83.23 ± 9.96<br>n = 21	68.85 ± 9.8<br>n = 25	76.58 ± 8.34<br>n = 22
IPSC rise time (ms)	1.03 ± 0.1<br>n = 20	1.69 ± 0.33<br>n = 18	1.17 ± 0.1<br>n = 23	1.25 ± 0.06<br>n = 22
mIPSC amplitude (pA)	47.20 ± 5.48<br>n = 18	28.11 ± 4.28<br>n = 22	44.07 ± 4.14<br>n = 22	22.39 ± 2.28<br>n = 20
mIPSC charge (fC)	806.8 ± 84.65<br>n = 18	499 ± 84.86<br>n = 22	798.6 ± 73.32<br>n = 22	420.6 ± 48.36<br>n = 20
mIPSC decay time (ms)	22.00 ± 1.19<br>n = 16	26.12 ± 2.56<br>n = 15	21.07 ± 1.69<br>n = 22	19.80 ± 1.06<br>n = 15
mIPSC rise time (ms)	0.92 ± 005<br>n = 16	0.67 ± 0.06<br>n = 15	0.66 ± 0.04<br>n = 22	0.89 ± 0.09<br>n = 15
mIPSC freq.	2.62 ± 0.61<br>n = 18	1.60 ± 0.34<br>n = 22	1.24 ± 0.41<br>n = 22	1.52 ± 0.29<br>n = 19
RRP (nC)	2.75 ± 0.43<br>n = 14	0.71 ± 0.18<br>n = 14	1.91 ± 0.30<br>n = 18	0.87 ± 0.13<br>n = 20
Num. vesicles	3849 ± 782.7<br>n = 14	2090 ± 799.3<br>n = 13	3043 ± 710.4<br>n = 18	2468 ± 366.6<br>n = 18
PPR_{100ms}	0.71 ± 0.06<br>n = 17			0.75 ± 0.07<br>n = 22
PPR_{50ms}		0.79 ± 0.07<br>n = 24	0.87 ± 0.07<br>n = 24	0.83 ± 0.07<br>n = 17
$IPSC_{suc}$ charge (nC)	0.32 ± 0.07<br>n = 14	0.16 ± 0.07<br>n = 14	0.25 ± 0.11<br>n = 18	0.26 ± 0.05<br>n = 19
Pvr (%)	14.21 ± 2.56<br>n = 14	27.13 ± 6.82<br>n = 14	10.19 ± 3.01<br>n = 18	32.11 ± 5.53<br>n = 19
Soma area (µm^2)	176.3 ± 14.15<br>n = 22	164.2 ± 16.85<br>n = 14	122.8 ± 8.44<br>n = 22	120.6 ± 11.35<br>n = 23
Dendr. Length (µm)	1341 ± 145<br>n = 22	805 ± 105<br>n = 14	1060 ± 107<br>n = 22	752 ± 69<br>n = 23
Dendr. Thickness (µm)	1.7 ± 0.11<br>n = 22	1.46 ±0.14<br>n = 14	0.96 ± 0.06<br>n = 22	1.11 ± 0.07<br>n = 23
VGAT synapses	212 ± 36<br>n = 22	35 ± 6<br>n = 14	229 ± 23<br>n = 22	157 ± 33<br>n = 23
