# Supplementary figures and images for: GABAergic synaptic components are largely preserved across human and mouse neuronal models
Source: Front Cell Neurosci. 2025 May 2;19:1588894. doi: 10.3389/fncel.2025.1588894 (PMC12082711; doi:10.3389/fncel.2025.1588894)

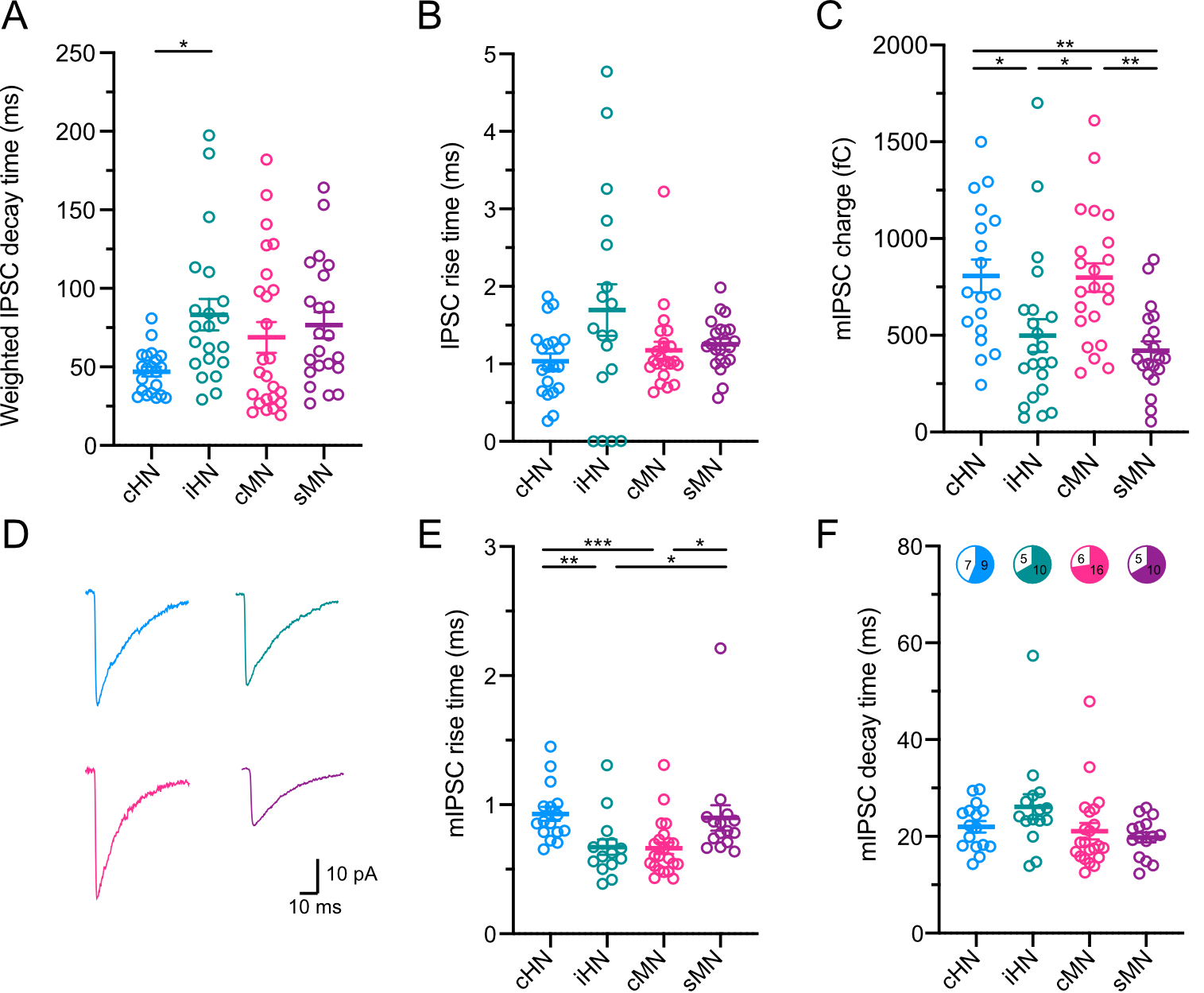

Supplement: Supplementary Figure 1 — Kinetic characterization of GABAergic synaptic transmission. (A) Scatter-plot of IPSC decay times. (B) Scatter-plot showing mIPSC rise times. (C) Scatter-plot showing mean mIPSC charges. (D) Representative averaged traces of mIPSC. (E) Scatter-plot showing mIPSC rise times. (F) Scatter-plot showing mIPSC decay times. Pie-plot insets represent number of neurons fitting to single (solid) or double (empty) exponential decay function. Data shown as mean ± SEM. Kruskal-Wallis test: *p ≤ 0.05, **p ≤ 0.01, ***p ≤ 0.001, and ****p ≤ 0.0001. [file Image_1.jpg]
